# Detection of the Disorders of Glycerophospholipids and Amino Acids Metabolism in Lung Tissue From Male COPD Patients

**DOI:** 10.3389/fmolb.2022.839259

**Published:** 2022-03-03

**Authors:** Qian Huang, Xiaojie Wu, Yiya Gu, Ting Wang, Yuan Zhan, Jinkun Chen, Zhilin Zeng, Yongman Lv, Jianping Zhao, Jungang Xie

**Affiliations:** ^1^ Department of Respiratory and Critical Care Medicine, National Clinical Research Center of Respiratory Disease, Key Laboratory of Pulmonary Diseases of Health Ministry, Tongji Hospital, Tongji Medical College, Huazhong University of Science and Technology, Wuhan, China; ^2^ Department of Respiratory and Critical Care Medicine, Wuhan Hospital of Traditional Chinese and Western Medicine, Wuhan, China; ^3^ Department of Science, Western University, London, ON, Canada; ^4^ Department and Institute of Infectious Disease, Tongji Hospital, Tongji Medical College, Huazhong University of Science and Technology, Wuhan, China; ^5^ Health Management Center, Tongji Hospital, Tongji Medical College, Huazhong University of Science and Technology, Wuhan, China

**Keywords:** COPD, lung tissue metabolomics, plasma metabolomics, glycerophospholipids, amino acids, biomarkers

## Abstract

**Background:** At present, few studies have reported the metabolic profiles of lung tissue in patients with COPD. Our study attempted to analyze the lung metabolome in male COPD patients and to screen the overlapping biomarkers of the lung and plasma metabolomes.

**Methods:** We performed untargeted metabolomic analysis of normal lung tissue from two independent sets (the discovery set: 20 male COPD patients and 20 controls and the replication set: 47 male COPD patients and 27 controls) and of plasma samples from 80 male subjects containing 40 COPD patients and 40 controls.

**Results:** We found glycerophospholipids (GPs) and Amino acids were the primary classes of differential metabolites between male COPD patients and controls. The disorders of GPs metabolism and the valine, leucine and isoleucine biosynthesis metabolism pathways were identified in lung discovery set and then also validated in the lung replication set. Combining lung tissue and plasma metabolome, Phytosphingosine and l-tryptophan were two overlapping metabolites biomarkers. Binary logistic regression suggested that phytosphingosine together with l-tryptophan was closely associated with male COPD and showed strong diagnostic power with an AUC of 0.911 (95% CI: 0.8460-0.9765).

**Conclusion:** Our study revealed the metabolic perturbations of lung tissues from male COPD patients. The detected disorders of GPs and amino acids may provide an insight into the pathological mechanism of COPD. Phytosphingosine and l-tryptophan were two novel metabolic biomarkers for differentiating COPD patients and controls.

## Introduction

Chronic obstructive pulmonary disease (COPD) is a chronic inflammatory disease of the airway. With high mortality and morbidity, it has caused a huge global burden and would be the third leading cause of death worldwide (Quaderi and Hurst, 2018). COPD is a heterogeneous disease with complicated pathogenic mechanism which has not been fully elucidated. In prior researches, whole genome sequencing, transcriptomics and proteomics have been widely utilized for biomarker screening and molecular mechanism research (Kan et al., 2017). A large number of genes and protein molecules have been determined to be associated with COPD (Stockley et al., 2019). Metabolites have also been implicated in various pathological processes in recent years (Johnson et al., 2016). Metabolites associated with diseases not only work as the promising biomarker for disease screening, but also provide an insight into the pathogenesis of disease (Arakaki et al., 2008). Therefore, researches identifying promising metabolite biomarkers to screen patients with COPD and to elucidate the pathogenic mechanisms associated with metabolites are warranted.

Metabolomic, the profiling of metabolites, is an emerging approach to identifying metabolite biomarkers and discovering the perturbed metabolic pathways that may help to elucidate the mechanisms governing COPD development (Johnson et al., 2016). Untargeted metabolomics can measure the widest array of metabolites in a clinical sample and has been implemented in recent years to uncover changes in metabolic pathways and identify novel biomarkers in many different diseases (Chen et al., 2019; Puchalska et al., 2019; Bunning et al., 2020; Liu et al., 2020; Thome et al., 2020). Metabolic disorders have also been identified in COPD patients. Previous studies have focused on the metabolism profiling of plasma (Paige et al., 2011), exhaled breath condensate (EBC) (Kilk et al., 2018), bronchoalveolar lavage fluid (BALF) (Yu et al., 2019), and induced sputum (Telenga et al., 2014) of patients or plasma and serum from the mouse model of emphysema or bronchitis caused by cigarette smoke (CS) (Cruickshank-Quinn et al., 2014; Ren et al., 2016). It is worth noting that samples in those studies could not reflect the metabolism of lung directly and the differential metabolites have not been validated in another independent set. Lung tissue from COPD patients is a more direct reflection of metabolism of the lung, however, has been rarely study. Though a recent study with a limited sample size reported the metabolomic of para-cancer tissue from lung cancer patients with COPD (Li et al., 2020), more studies with normal tissue away from lung nodules with a larger sample size are necessary.

Considering that lung tissue is a direct reflection of the metabolism of lung cells and has been rarely explored and the prevalence of COPD in men is more than twice that of women (Fang et al., 2018), we collected two independent sets of lung tissues from male COPD patients and controls to perform untargeted metabolomics analysis. We attempted to screen and further validate the differential metabolites associated with male COPD and disordered metabolic pathways. Furthermore, we also performed plasma metabolomic analysis to determine the overlapping metabolites between plasma and lung tissue; these metabolites would be more precise biomarkers to effectively discern COPD patients from controls.

## Materials and Methods

### Patient Sample Collection

All samples were collected from Tongji Hospital of Tongji Medical College, Huazhong University of Science and Technology, Wuhan, Hubei, China. we obtained approval from the ethics committee of Tongji Hospital, Huazhong University of Science and Technology, Wuhan, Hubei, China, and each subject signed an informed consent form before the collection of samples.

For the lung discovery set, lung tissues were collected from 20 male patients with COPD and 20 controls. For the lung validation set, lung tissues were collected from 47 male patients with COPD and 27 controls. All subjects with lung tissue collected had undergone a surgical operation for lung nodules after undergoing pulmonary function testing. Specimens were dissected at a distance of more than 5 cm from the tumor. Furthermore, the collection of plasma samples was also implemented in 40 patients and 40 controls (20 patients and 20 controls were the same subjects as subjects in the lung discovery set, while the other 20 patients and 20 controls were collected from a follow-up cohort study in Hankou, Wuhan, China). In our study, COPD was diagnosed according to the global initiative for chronic obstructive pulmonary disease (GOLD). Subjects with respiratory infection or other chronic pulmonary diseases (asthmas, bronchiectasis and interstitial lung diseases (ILDs)), having systemic steroid use within the previous 4 weeks, or who had a history of other cancers were excluded. All samples collected were stored in a freezer at a temperature of −80°C until use.

### Sample Pretreatment for LC-MS

50 mg of each lung tissue was weighted out and 200 μL extracting solution (extracting solution is 80% methanol containing l-Phenylalanine-D8, l-Valine-D8, Taurine (1, 2-13C2), 2-Chloro-l-phenylalanine as internal standard. The concentrations of internal standards were 5 μg/ml) was added to it. The mixture was homogenized six times, 1 min at 60 Hz for each time. Then the mixture was centrifugalized at 10,000 rpm, 4°C for 15 min. The supernatant was transferred to sampler vials for detected. 100 μL of each plasma sample was taken out and 400 μL extracting solution was added to it. The mixture was mixed by vortex mixer for 5 min and centrifuged at 13,000 rpm, 4°C for 15 min. The supernatant was transferred to sampler vials for detected. An in-house quality control (QC) was prepared by mixing equal volume of each sample.

### LC-MS Analysis

A 1290 InfinityⅡUHPLC system coupled with a 6545 UHD and Accurate-Mass Q-TOF/MS was used for LC-MS analysis (Agilent Technologies, CA, USA). Considering that different chromatographic columns have different sensitivities to detect metabolites, two different chromatographic columns have been used to screen more metabolites associated with COPD in our study. And the two chromatographic columns used were Waters XBridge UPLC BEH T3 (2.1 × 100 mm, 2.5 μm) and Waters XBridge UPLC BEH Amide (2.1 × 100 mm, 2.5 μm). For Waters XBridge UPLC BEH T3: Mobile phase A was 0.1% formic acid. Mobile phase B consisted of acetonitrile solution with 0.1% formic acid. The flow rate was 0.4 ml/min, the column temperature was 25°C and the injection volume is 4 μL. Gradient elution condition was set as follows: 0-2 min, 2% B; 2-13 min, 2-98% B; 13-15 min, 98% B. Post time was set as 6 min for system balance. For Waters XBridge UPLC BEH Amide: Mobile phase A was composed of 0.1% formic acid and 10 mM ammonium formate. Mobile phase B consisted of acetonitrile solution with 0.1% formic acid. The flow rate was 0.4 ml/min, the column temperature was 25°C and the injection volume was 4 μL. The gradient elution was set as follows: 0-1 min, 95% B; 1-3 min, 95–85% B; 3-13 min, 85–60% B. Post time was set as 5 min for system balance.

Mass spectrometry was operated in both positive and negative ion modes. The parameters optimized were as follows: Capillary voltage in positive and negative mode was 4 and 3.5 kV, respectively, drying gas flow was 101/min, gas temperature was 325°C, nebulizer pressure was 20 psig, fragmentor voltage was 120 V, skimmer voltage was 45 V and mass range was 50–1100 m/z. Reference ions were used during MS acquisition process to ensure mass accuracy. Reference ions in positive ion mode: 121.0509, 922.0098. Negative ion mode: 112.9856, 1033.9981.

Acquired raw data were converted to the mz.data format by Agilent Masshunter Qualitative Analysis B.07.00 software (Agilent Technologies, USA). In the R software platform, the XCMS program was used for peak identification, retention time correction, and automatic integration pretreatment. Next, the data were subjected to internal standard normalization and weight normalization. Visualization matrices containing the sample name, m/z-RT pair and peak area were obtained. Putative metabolite annotation was performed using the Human Metabolome Database (HMDB), METLIN and PUBCHEM Database, and output matched to an in-house accurate mass/retention time library of reference standards (Wishart et al., 2013; Naz et al., 2017).

### Statistical Analysis

The Baseline characteristics of the study subjects were collected and described. Student’s t-test for continuous variables and a chi-square test for categorical variables were implemented to uncover the differences in fundamental characteristics of study subjects. With *p*-values < 0.05 serving as the threshold for significance, statistical analyses were performed using GraphPad Prism 8.0.1.

For metabolic profiling data, multivariable analysis, orthogonal partial least squares-discriminant analysis (OPLS-DA), was conducted using SIMCA-P software (version 14.1, Umetrics, Umea Sweden). OPLS-DA is a supervised multivariable analysis technique. Variable importance in projection (VIP) scores of OPLS-DA above 1.0 allied with Student’s t-test (The Benjamini–Hochberg false discovery rate (FDR) method was used for multiple testing adjustment, FDR <0.05) were commonly utilized to identify metabolites with differential expression between patients with COPD and controls. Furthermore, the cumulative modeled variation in the X and Y matrix (*R* ^2^ X and *R* ^2^ Y) and the cross-validated predictive ability Q^2^ (cum) values were used to assess the quality of the models. ANOVA of the cross-validated residuals (CV-ANOVA, *p* < 0.05) was performed to evaluate the reliability of the OPLS-DA model.

Logistic regression analysis was employed to combine the predictive ability of overlapping metabolite biomarkers, which was subsequently evaluated using receiver operating curve (ROC) plots (forward stepwise selection with a significance threshold of 0.10 for removal). Pathway analysis was performed using the analysis platform Metaboanalyst (http://www.metaboanalyst.ca/MetaboAnalyst/). Volcano plots, expression heatmaps and correlation heatmaps were generated using R version 4.0.2 (https://www.r-project.org/). Venn diagram Venn diagrams were drawn on the websites (http://bioinformatics.psb.ugent.be/webtools/Venn/). All other statistical diagrams were drawn using GraphPad Prism 8.0.1.

## Results

### Basic Characteristics of Study Subjects

Our study contained three independent sets with a total of 114 lung tissues and 80 plasma samples. The discovery set included 20 male COPD patients and 20 controls; the replication set included 47 male COPD patients and 27 controls. The plasma set consisted of male 40 COPD patients and 40 controls. The basic characteristics of the study subjects in the three sets are presented in Table 1. No matter in which set, there were no significant differences in sex, age, BMI or smoking status between COPD patients and controls, while there were significant differences in FEV1% predicted and FEV1/FVC% except for a little difference of age in discovery set.

**TABLE 1:** Fundamental Characteristics of Study Subjects.

	Discovery set (lung tissue)			Plasma set			Replication set (lung tissue)		
	control (*n* = 20)	COPD (*n* = 20)	*p* value	control (*n* = 40)	COPD (*n* = 40)	*p* value	control (*n* = 27)	COPD (*n* = 47)	*p* value
Age (Y)	56 (50–63)	61 (56–65)	*p* = 0.023	59 (54–65)	62 (57–68)	*p* = 0.11	62.3 (59.4–65.3)	66.6 (57.7–75.5)	*p* = 0.573
Gender (Man/Woman)	20/0	20/0	NA	40/0	40/0	NA	27/0	47/0	NA
Non-smokers/ex-smokers/smokers	3/3/14	3/7/10	*p* = 0.398	3/9/28	3/16/21	*p* = 0.327	10/5/17	7/10/34	*p* = 0.432
BMI	23 (21–26)	23 (21–26)	*p* = 0.9	24 (19–28)	23 (19–28)	*p* = 0.44	27 (26–28)	26 (25–27)	*p* = 0.356
FEV1/FVC%	79 (66–92)	61 (54–68)	*p* < 0.001	77 (71–82)	55 (43–67)	*p* < 0.001	77 (74–81)	64 (56–68)	*p* < 0.001
FVC	3.7(3.3–4.2)	3.4 (3.1–4.2)	*p* = 0.45	3.7 (3.5–4.2)	3.5 (3.2–4.0)	*p* = 0.07	3.6 (3.2–4.3)	3.8 (3.3–4.3)	*p* = 0.833
FVC% predicted	84.5(80.4–97.6)	87.1 (79.6–100.2)	*p* = 0.84	90.8 (83.5–100.9)	89.4 (79.6–100.5)	*p* = 0.125	102.0 (93.0-110.5)	104.0 (93.3–120.6)	*p* = 0.624
FEV1	2.9 (2.6–3.4)	2.1 (1.8–2.6)	*p* < 0.001	2.9 (2.7–3.3)	2.1 (1.6–2.5)	*p* < 0.001	2.8 (2.5–3.2)	2.4 (1.9–2.7)	*p* < 0.001
FEV1% predicted	91 (77–105)	71 (58–85)	*p* < 0.001	102 (85–119)	67 (45–88)	*p* < 0.001	100 (91–107)	80 (63–97)	*p* < 0.001

Data are presented as n or s n or median (interquartile range). BMI, body mass index; COPD, chronic obstructive pulmonary disease; FEV1: forced expiratory volume in 1 s; FVC: forced vital capacity; NA, not available.

### Metabolic Profiles of Lung Tissue and Plasma in COPD Patients

We first performed nontargeted metabolomics analysis on the discovery lung set, 2207 and 2227 features were detected with the T3 and amide methods, respectively. To expand the sample size to further verify the perturbations of lung tissue metabolism and to test the reproducibility of the data, an independent lung replication set was subsequently incorporated into the analysis. Next, 1357 and 2464 features were calculated in the T3 and amide methods of the replication set, respectively. Finally, to screen the overlapping metabolite biomarkers in the plasma and lung metabolome, we also included another plasma set for analysis. A total of 4426 features were discovered through the T3 (2457) and amide (1969) methods.

**FIGURE 1:**
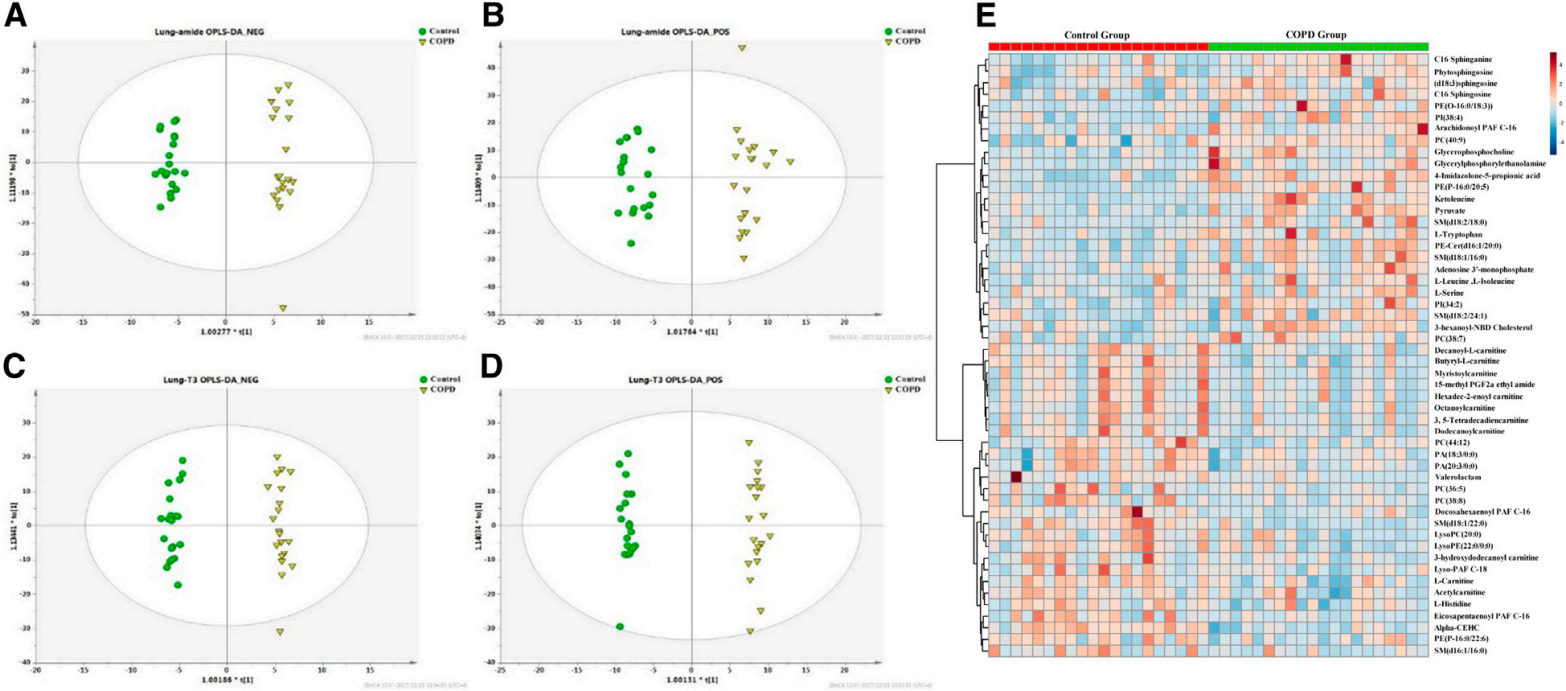
The OPLS-DA model for classifying COPD patients versus controls displayed significant group deviation in Discovery set. **(A)** The score plot of the amide method in negative mode. **(B)** The score plot of the amide method in positive mode. **(C)** The score plot of the T3 method in negative. **(D)** The score plot of the T3 method in positive mode. **(E)** The heatmap showed 52 differentially abundant metabolites of the COPD patients compared with the controls in discovery set, the red color indicated higher expression level in COPD and the blue color indicated lower expression level in COPD. OPLS-DA, orthogonal partial least squares-discriminant analysis. COPD, chronic obstructive pulmonary disease.

Next, we performed orthogonal partial least squares-discriminant analysis (OPLS-DA) on the data of the discovery set. OPLS-DA score plots exhibited a good separation between COPD patients and controls, with a *p*-value less than 0.05 in the CV-ANOVA test suggesting the reliability of the models (Figures 1A–D). The same phenomenon was observed in both the replication and plasma sets. The permutation tests were carried out in all (O)PLS-DA models of the three sets, and the results showed that the models were reliable without overfitting (Table 2).

**TABLE 2:** (O)PLS-DA models’ parameters for three independent set.

		Discovery lung set				Plasma set				Replication set			
				OPLS-DA^d^				OPLS-DA^d^				PLS-DA	
		Amide-NEG	Amide-POS	T3-NEG	T3-POS	Amide-NEG	Amide-POS	T3-NEG	T3-POS	Amide-NEG	Amie-POS	T3-NEG	T3-POS
R2X(cum)a		0.358	0.282	0.378	0.37	0.221	0.296	0.378	0.37	0.429	0.398	0.432	0.372
R2Y(cum)a		0.986	0.956	0.973	0.996	0.777	0.918	0.973	0.362	0346	0.264	0.288	0.533
Q2(cum)c		0.584	0.584	0.788	0.846	0.242	0.695	0.788	0.846	0.22	0.152	0.184	0.221
CV-ANOVA (*p*-value)		7.394	3.086	1.720*e^19^	1.478*e^22^	0.002	1.537*e^15^	1.72*e^19^	1.477*e^22^	0.002	0.016	0.008	0.006
Permutation	R2Y(cum)a	0.959	0.898	0.970	0.981	0.733	0.849	0.898	0.975	0.243	0.247	0.249	0.442
Q2(cum)c		−0.357	−0.327	−0.396	−0.356	−0.362	−0.390	−0.438	−0.433	−0.121	−0.13	−0.143	−0.254

OPLS-DA, orthogonal partial least squares-discriminant analysis; ANOVA, of the cross-validated residuals; a R2X (cum) and R2Y (cum) are the cumulative modeled variations in the X and Y matrix, respectively; b Q2Y (cum) is the cumulative predicted variation in the Y matrix; c P, predictive component; d O, orthogonal component.

### Differential Metabolites Associated With COPD in Both the Discovery and Replication Sets

**FIGURE 2:**
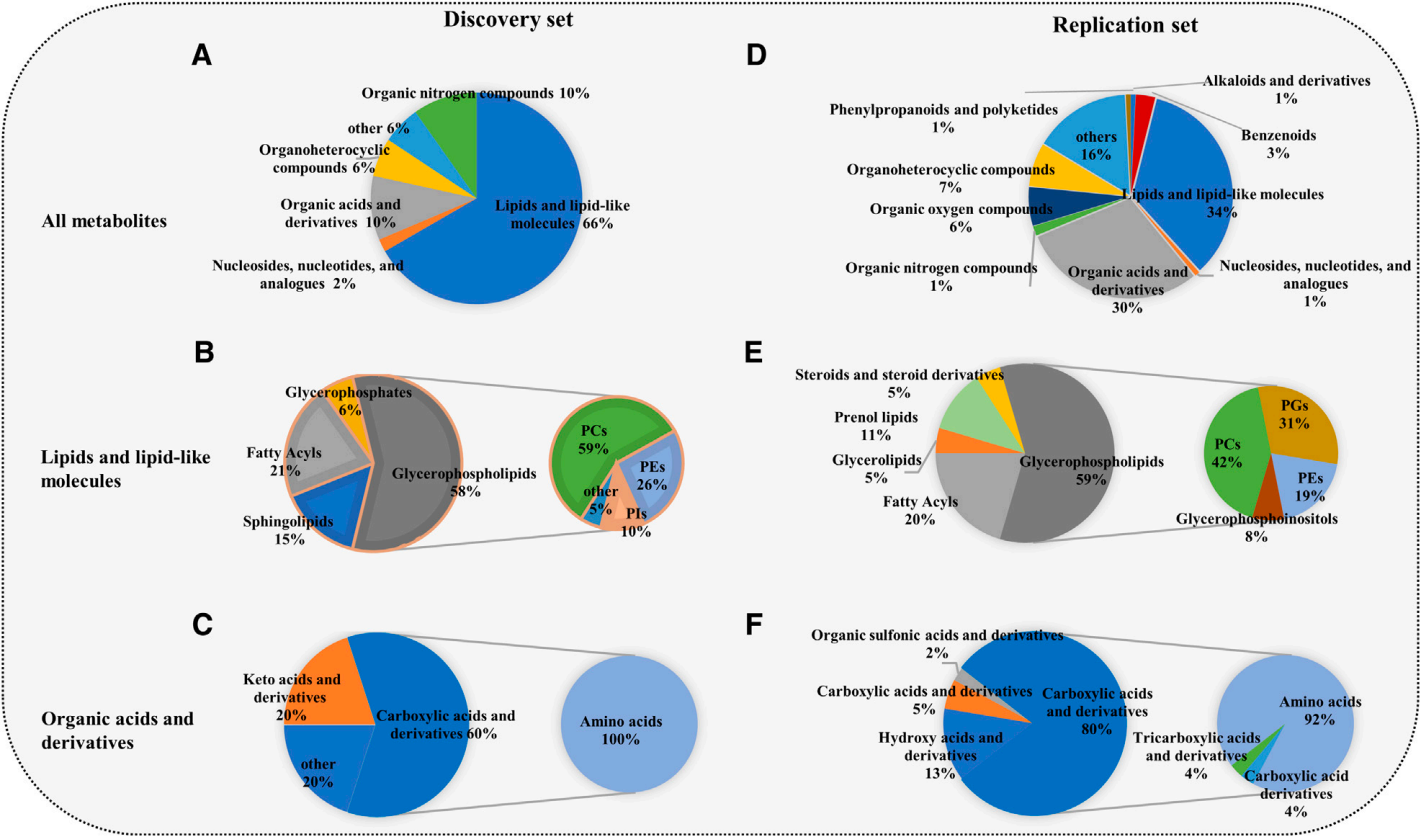
Composition of the differential metabolites in the discovery set and replication set. **(A,D)** The superclasses of all metabolites. **(B,E)** Lipids and lipid-like molecules and organic acids and their derivatives were the most common superclasses. **(C,F)** Glycerophospholipids and amino acids were the main classes. The primary pie shows the composition of lipids and lipid-like molecules and organic acids and derivatives, and the secondary pie displays the component category of glycerophospholipids and amino acids. The superclass, class and subclass of metabolites were acquired from the Human Metabolome Database. GPs, glycerophospholipids; PCs, glycerophosphocholines; PGs, glycerophosphoglycerols; PIs, glycerophosphoinositols; PEs, glycerophosphoethanolamines; amino acids: amino acids, peptides and analogs.

The union of the differential compounds in T3 and amide methods serves as the final metabolites associated with COPD. In the discovery set, 52 significantly different metabolites between COPD patients and controls were identified. The heatmap showed 52 differential metabolites (Figure 1E). The main superclasses (Figure 2A) of 52 metabolites were 66% lipids and lipid-like molecules (primarily containing 58% glycerophospholipids (Figure 2B), 21% fatty acyls and 15% sphingolipids), 10% organic acids and derivatives (60% Carboxylic acids and derivatives) (Figure 2C).

**FIGURE 3:**
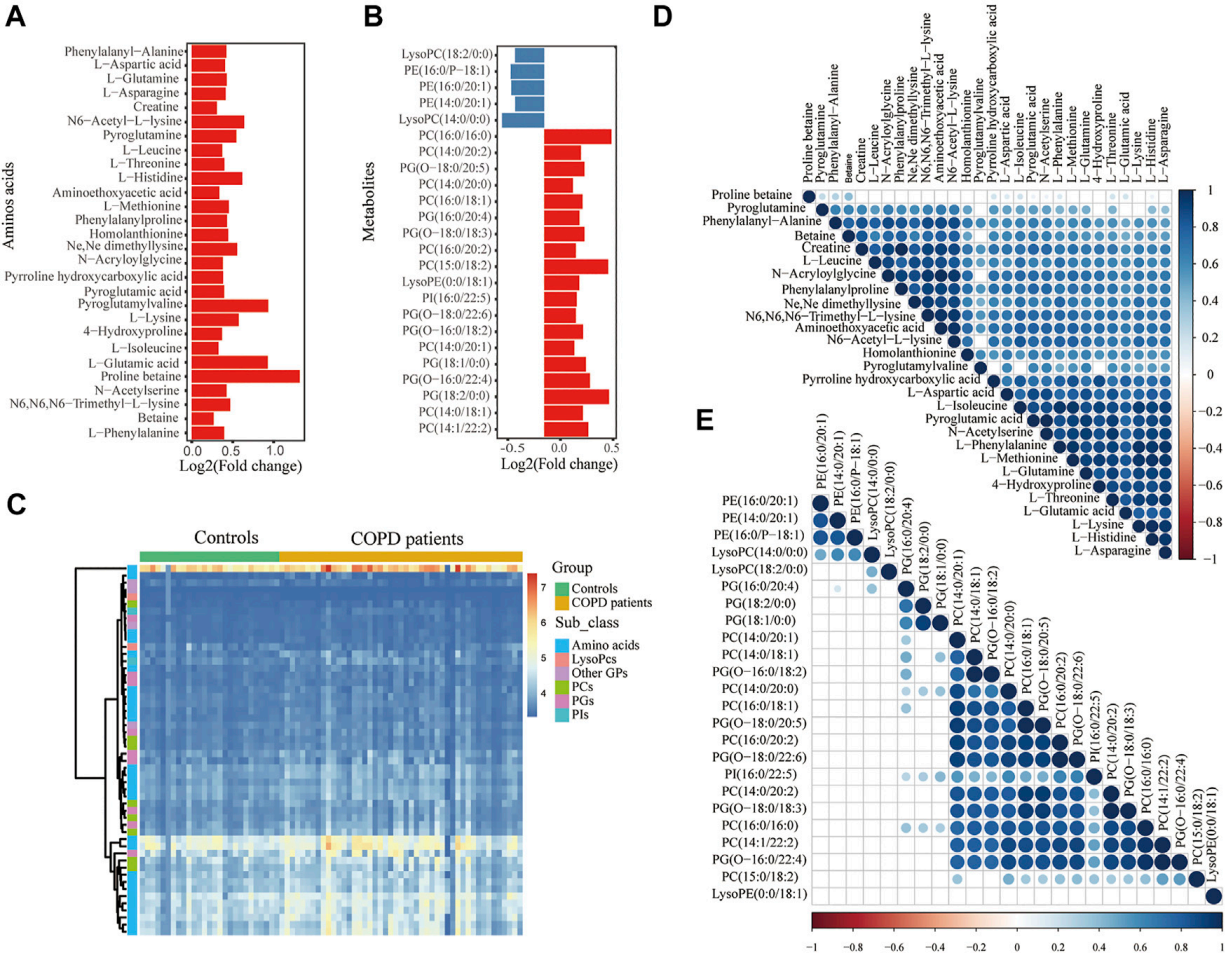
Significantly changed metabolism of GPs and amino acids in COPD in the replication set. Histogram of significant metabolites of glycerophospholipids **(A)** and amino acids **(B)**. The *y*-axis shows the metabolites, while the *x*-axis shows the value of log2 (fold change). **(C)** The heatmap shows differential GPs and amino acids of the COPD patients compared with the controls. Spearman correlation heatmap of **(D)** amino acids and **(E)** glycerophospholipids. The larger the circle and the darker the color indicate the greater the correlation coefficient. The circle is not displayed when the correlation is not significant. GPs, glycerophospholipids; PCs, glycerophosphocholines; PGs, glycerophosphoglycerols; PIs, glycerophosphoinositols; PEs, glycerophosphoethanolamines; amino acids: amino acids, peptides and analogs.

Unsurprisingly, the replication set obtained the same findings in the discovery set. A total of 371 metabolites associated with COPD were finally identified. Among these metabolites, lipids and lipid-like molecules (34%) were the primary superclass, other superclasses mainly contained organic acids and derivatives (30%) and organonitrogen compounds (6%) (Figure 2D). Glycerophospholipids (59%) were the main class of lipids and lipid-like molecules (Figure 2E). Other classes were 20% fatty acyls and 11% prenol lipids. Eighty percent of organic acids and their derivatives were carboxylic acids and derivatives (Figure 2F). In the replication set, 28 subclasses of amino acids and 24 subclasses of glycerophospholipids were significantly changed in COPD. Among them, notably, almost all amino acids were elevated (Figure 3A). 19 glycerophospholipids were upregulated, while only five of 24 were reduced (Figure 3B). The heatmap showed the expression of glycerophospholipids and amino acids (Figure 3C). In COPD patients, spearman correlation analysis showed that both different subclasses of glycerophospholipids and amino acids presented a positive correlation (Figures 3D,E). In addition, we performed Spearman correlation analysis on the relative expression of glycerophospholipids and amino acids with some critical clinical indicators. However, the correlations between these groups were not highly significant (Supplementary Figure S1 and Supplementary Table S1).

### Pathway Analysis

**FIGURE 4:**
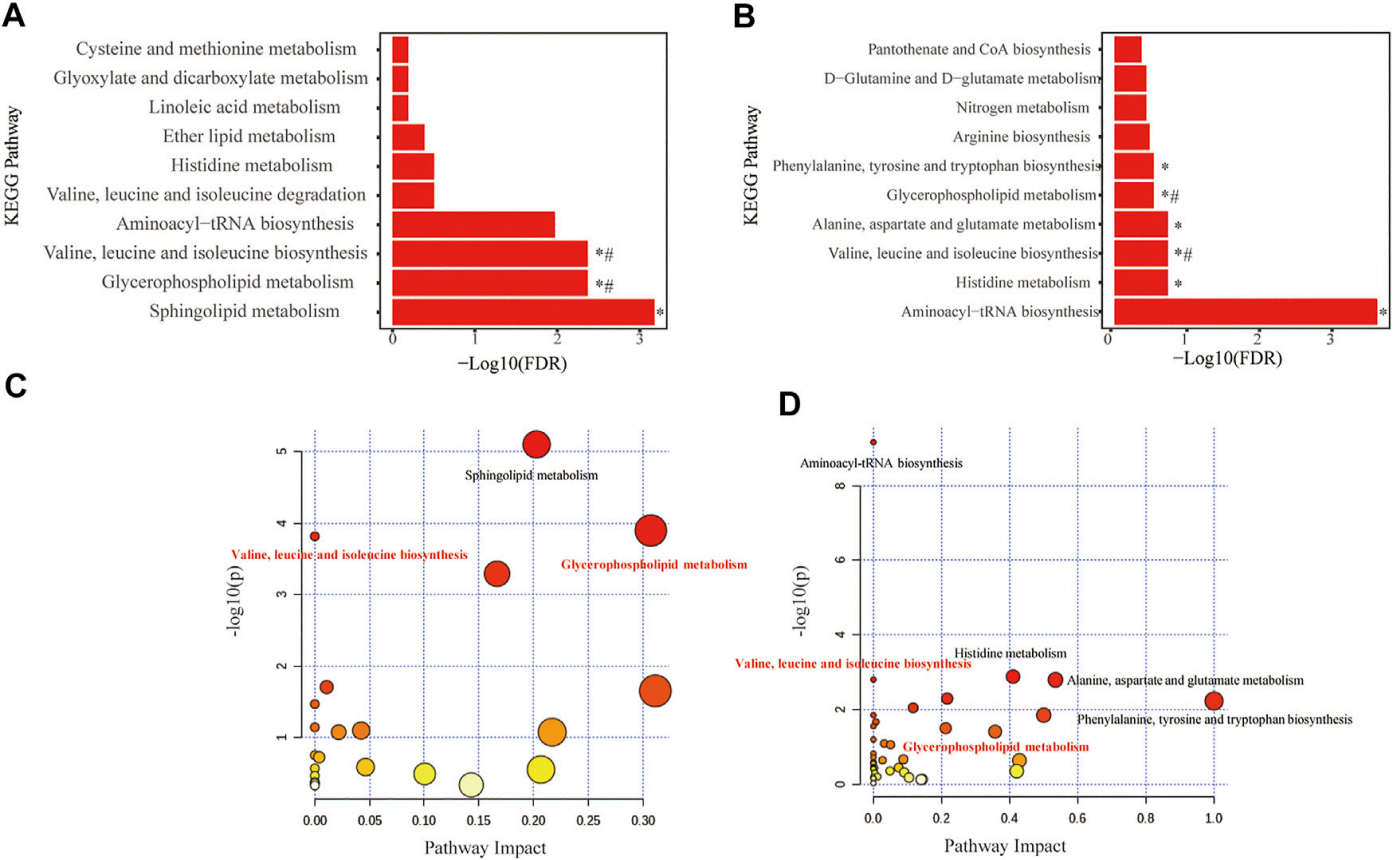
Metabolome view of pathway impact analysis obtained from differential metabolites in COPD. Significantly altered pathways in discovery set **(A,B)** and in replication set **(C,D)**. The color and size of each circle are based on *p*-values (yellow: higher *p*-values and red: lower *p*-values) and pathway impact values (the larger the circle, the higher the impact score) calculated from the topological analysis, respectively. Pathways were considered significantly changed if adjusted *p*-value (FDR) < 0.05. *Significantly changed pathways in the recovery or replication set. #Significantly changed pathways in both the recovery and replication sets. FDR, False discovery rate.

Fifty-two distinguished metabolites from the discovery set were first utilized to perform pathway analysis. Consequently, multiple metabolic pathways, including sphingolipid metabolism, glycerophospholipid metabolism and valine, leucine and isoleucine biosynthesis pathways, were significantly (*p* < 0.05 with FDR <0.1) perturbed (Figure 4A,B). For validation, pathway analysis of the validation set was also performed. Six metabolic pathways with perturbations were eventually detected, and two of them (namely, glycerophospholipid metabolism and the valine, leucine and isoleucine biosynthesis pathways) were consistent with the discovery set (Figures 4C,D). Comprehensive information on the detected pathways was provided in Tables 3, 4.

**TABLE 3:** Pathway analysis showed that metabolic pathways significantly altered in COPD patients against Controls in Discovery set.

	Recovery set				
	Total cmpd^a^	Hits^b^	Raw p^c^	FDR^d^	Impact
Sphingolipid metabolism	21	5	0.00000791	0.0006403	0.20284
Glycerophospholipid metabolism	36	5	0.0001264	0.0042698	0.30734
Valine, leucine and isoleucine biosynthesis	8	3	0.00015249	0.0042698	0
Aminoacyl-tRNA biosynthesis	48	5	0.00051012	0.010713	0.16667
Valine, leucine and isoleucine degradation	40	3	0.019719	0.31195	0.01084
Histidine metabolism	16	2	0.022282	0.31195	0.31147
Ether lipid metabolism	20	2	0.034034	0.40841	0

d COPD, chronic obstructive pulmonary disease; ^a^Total Cmpd: total number of compounds in the pathway; ^b^Hit, actually matched number from the data; ^c^Raw p, *p* value calculated from the enrichment analysis; FDR, *p* value adjusted using False Discovery Rate.

**TABLE 4:** Pathway analysis of COPD patients against Controls in Replication set.

	Replication set				
	Total Cmpd^a^	Hits^b^	Raw p^c^	FDR^d^	Impact
Aminoacyl-tRNA biosynthesis	48	13	6.77E-10	5.69E-08	0
Histidine metabolism	16	4	0.001311	0.033588	0.40983
Valine, leucine and isoleucine biosynthesis	8	3	0.001579	0.033588	0
Alanine, aspartate and glutamate metabolism	28	5	0.001599	0.033588	0.53446
Glycerophospholipid metabolism	36	5	0.005047	0.082213	0.21631
Phenylalanine, tyrosine and tryptophan biosynthesis	4	2	0.005872	0.082213	1
Arginine biosynthesis	14	3	0.00895	0.1074	0.11675
Nitrogen metabolism	6	2	0.014083	0.13144	0
D-Glutamine and D-glutamate metabolism	6	2	0.014083	0.13144	0.5
Pantothenate and CoA biosynthesis	19	3	0.021269	0.17866	0.00714
beta-Alanine metabolism	21	3	0.027902	0.21307	0
Arginine and proline metabolism	38	4	0.031523	0.22066	0.21189
Phenylalanine metabolism	10	2	0.038903	0.25137	0.35714

d COPD, chronic obstructive pulmonary disease; ^a^Total Cmpd, total number of compounds in the pathway; ^b^Hit,actually matched number from the data; ^c^Raw p, *p* value calculated from the enrichment analysis; FDR, *p* value adjusted using False Discovery Rate.

### Overlapping Candidate Metabolite Biomarkers in Lung Tissue and Plasma

**FIGURE 5:**
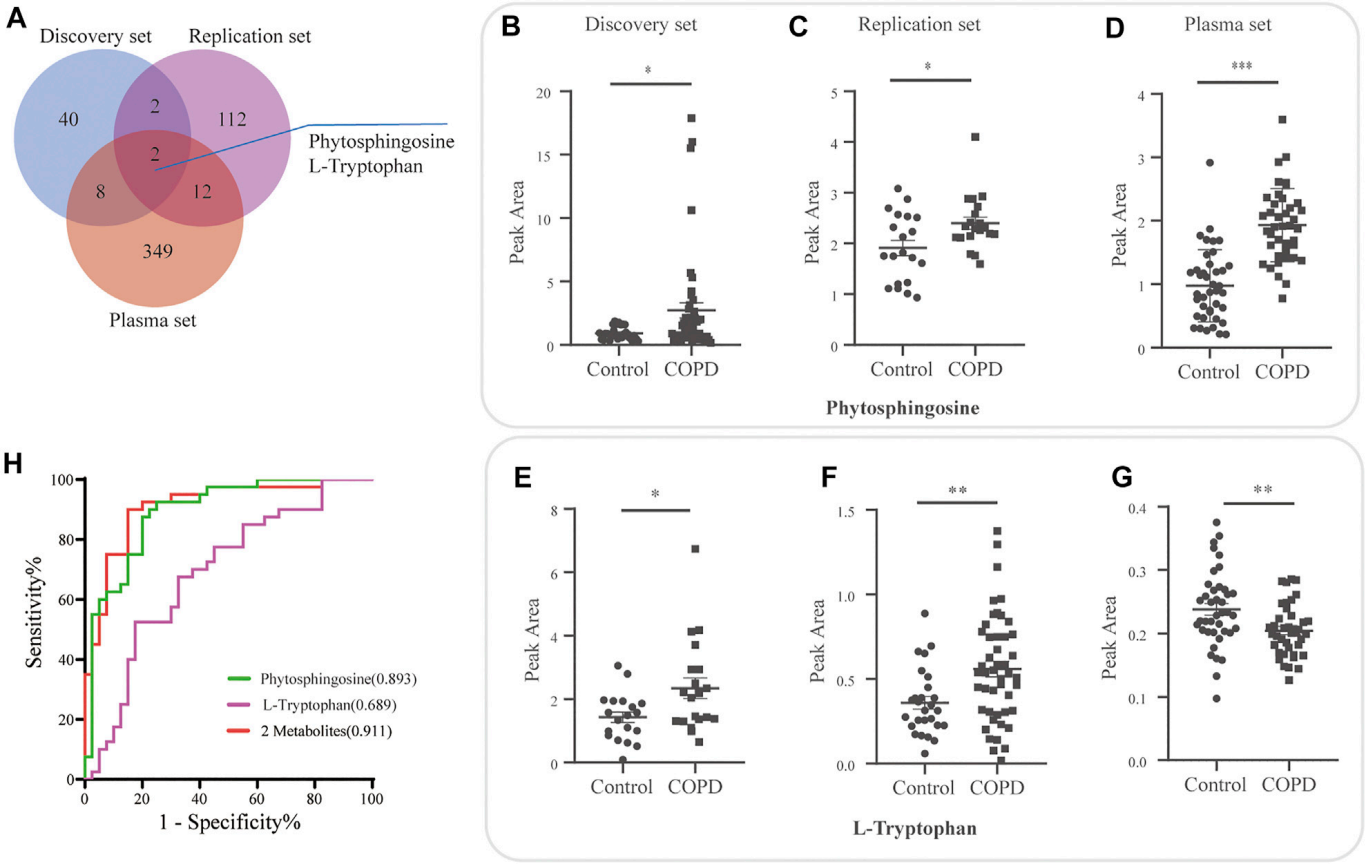
Overlapping candidate metabolite biomarkers in three sets. **(A)** Venn diagram demonstrating overlap and unique differential metabolites for the discovery, plasma and replication sets. Relative expression of phytosphingosine **(B–D)** and l-tryptophan **(E–G)** in the discovery, plasma and replication sets. **(H)** Evaluation of the diagnostic value of the overlapping metabolites by logistic regression model, and receiver operating curve (ROC) analysis and area under the curve (AUC).

For the plasma set, 134 differentially expressed metabolites were detected. A Venn diagram demonstrated two overlapping metabolites (phytosphingosine and l-tryptophan) in the discovery, plasma and replication sets (Figure 5A and Supplementary Table S2). The relative expression of phytosphingosine (Figures 5B–D) and l-tryptophan (Figures 5E–G) in three different sets was shown. The variable importance in projection (VIP) score, fold change (FC) and FDR of two overlapping metabolites were presented (Table 5).

**TABLE 5:** Overlapping metabolites in lung between Discovery set and replication set.

Lipid names	Discovery set			Replication set			Plasma set		
	VIP	FC (COPD/control)	FDR	VIP	FC (COPD/control)	FDR	VIP	FC(COPD/control)	FDR
Phytosphingosine	1.73	1.26	0.031	2.91	2.97	0.041	2.38	1.98	<0.0001
L-Tryptophan	1.87	1.64	0.031	1.13	1.55	0.032	1.72	0.86	0.008

FC, fold change; COPD, chronic obstructive pulmonary disease.

Subsequently, the diagnostic performance of the two candidates was evaluated by area under the curve (AUC) of the receiver operating curve (ROC) analysis (Figure 5H). Phytosphingosine exhibited a strong predictive performance (AUC = 0.8931, 95% CI: 0.8224-0.9639) with 87.5% sensitivity and 80% specificity. The AUC value of l-tryptophan was 0.69 (95% CI: 0.5711-0.8064) with 67.5% sensitivity and 67.5% specificity. Furthermore, the combination of two metabolites distinguished COPD and controls more effectively than any single biomarker candidate, which yielded a severe AUC of 0.911 (95% CI: 0.8460-0.9765) with 90.0% sensitivity and 85.0% specificity (Table 6).

**TABLE 6:** The diagnosis value of the overlapping metabolite biomarkers.

	Cut-point	Sensitivity (%)	Specificity (%)	AUC (95%CI)	*p* Value
Phytosphingosine	>1.324	87.5	80	0.8931(0.8224–0.9639)	<0.0001
L-Tryptophan	<0.213	67.5	67.5	0.69(0.5711–0.8064)	0.0037
2 Metabolites	>0.456	90.0	85.0	0.911(0.8460–0.9765)	<0.0001

SEN, sensitivity; SPE, specificity; AUC, area under the curve; 95%CI, 95% confidence interval. 2 metabolites, phytosphingosine and L-Tryptophan.

## Discussion

In our study, we performed untargeted metabolomics in lung tissue of male COPD patients. We found that several classes of metabolites, including glycerophospholipids (GPs), amino acids and fatty acyls, were abnormally expressed. Pathway analysis suggested that many abnormal metabolic pathways were associated with male COPD. Among these pathways, glycerophospholipid metabolism and valine, leucine and isoleucine biosynthesis pathways were disordered in both the discovery and replication set. Besides, we also tested another independent plasma set to screen the overlapping biomarkers between the lung tissue and plasma. We subsequently screened out two overlapping metabolites containing phytosphingosine and l-tryptophan, which showed a high diagnostic performance for discriminating early male COPD patients from controls.

Metabolomic profiling is an emerging tool for detecting differential metabolites, disrupted pathways related to diseases and potential biomarkers. It has also been evaluated in COPD patients. However, most of those studies focused on the metabolome of plasma, exhaled breath condensate (EBC), bronchoalveolar lavage fluid (BALF) or induced sputum in COPD patients and did not validate their findings with a replication set (Kilk et al., 2018); (Paige et al., 2011); (Ran et al., 2019); (Telenga et al., 2014); (Yu et al., 2019). Lung cells can reflect the metabolic profiling of patients with COPD more directly, while the normal lung tissue from COPD patients has rarely been calculated to date. Sex can influence the metabolism (Darst et al., 2019). A nationwide prevalence study revealed that the prevalence of COPD in men is more than twice that of women (Fang et al., 2018). Recent studies also reported that the difference in metabolic profiles between male and female COPD patients (Gillenwater et al., 2021); (Naz et al., 2017). Therefore, lung tissue from male COPD patients in two independent set were collected to perform metabolomic analysis: a discovery set to detect and a replication set to validate. What’s more, we also analyzed metabolomic profiling of plasma to screen the overlapping metabolites biomarkers.

**FIGURE 6:**
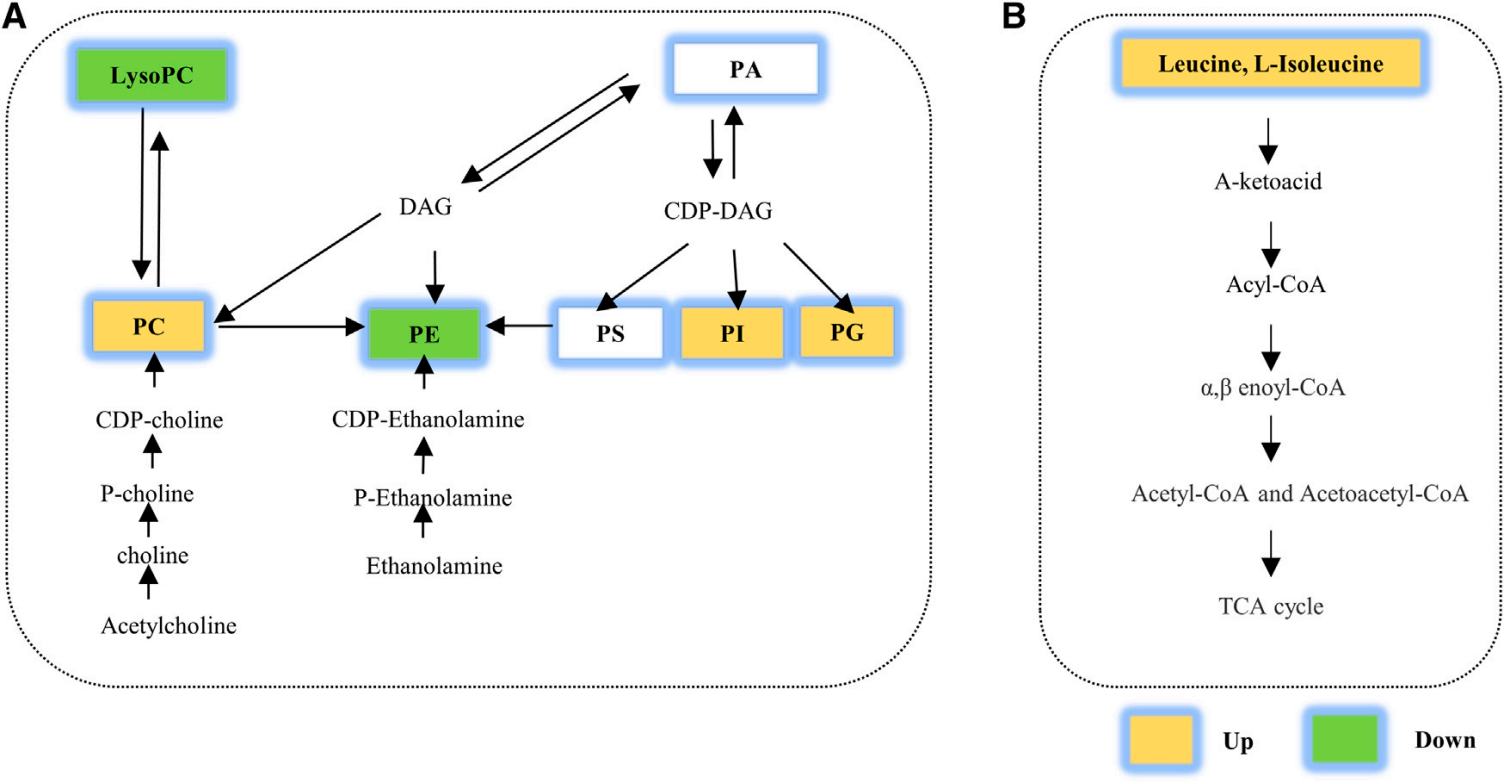
Several metabolic pathways with perturbations were calculated in our study. Perturbation of **(A)** glycerophospholipid metabolism and **(B)** amino acid metabolism. TCA cycle, tricarboxylic acid cycle; 5-HT, 5-hydroxytryptamine; 5-HIAA, 5-hydroxyindoleacetic acid. PE: phosphatidylethanolamines; DAG, diacylglycerol; PC, phosphatidylcholine; PS, phosphatidylserine; PG, glycerophosphoglycerols; PI, glycerophosphoinositols; PA, phosphatidic acid.

Glycerophospholipids (GPs), the most abundant phospholipids and one of the primary lipid types in cell membranes, consist of such lipids as phosphatidylethanolamine (PE), phosphatidylcholine (PC), phosphatidylglycerol (PG), phosphatidylserine (PS), phosphatidylinositol (PI), phosphatidic acid (PA), phosphatidylglycerophosphate (PGP) and CDP-diacylglycerol (CDP-DG) (Kimura et al., 2016). Predictably, these lipids play critical roles in lipid metabolism and health (van der Veen et al., 2017). The metabolism of GPs showed in Figure 6A. In recent studies, the perturbation of GPs has been observed in other diseases, such as osteoporosis (Mei et al., 2020; Bellissimo et al., 2021), type 2 diabetes (Lai et al., 2020; Schillemans et al., 2021), pleural effusion (Luo et al., 2020) or environmental microplastics (Zhao et al., 2021). In our study, three decreased PEs, nine increased PCs and eight elevated PEs were identified. How these abnormal GPs are involved in the pathological mechanism of COPD is currently unclear. A study reported that loss of PE induces mitochondrial dysfunction and oxidative stress (Heden et al., 2019). PE-deficient mice produced more H_2_O_2_, and mitochondrial PE deficiency is harmful to their metabolic and contractile functions, causing ventilatory failure and lethality. Another study showed that the increased PC levels may be caused by cigarette smoking and may participate in oxidative stress (Wang-Sattler et al., 2008; Li et al., 2016). In addition, it also reported that the destruction of the CDP-ethanolamine pathway for PE synthesis is related to cellular senescence (Wu et al., 2019) and the human T cell response could be triggered by PGs mediated by CD1b (Shahine et al., 2017). COPD is a chronic inflammatory disease associated with oxidative stress and cellular senescence (Birch et al., 2018). These evidences suggest that GPs may be related to COPD by participating in oxidative stress and inflammation, but this requires further verification.

In addition, both the discovery and validation sets showed perturbations in the class of amino acids by lung metabolomics analysis in our study. Meanwhile, disorders of the valine, leucine and isoleucine biosynthesis pathways have also been identified and validated (Figure 6B). Amino acids are essential nutrients and pivotal determinants of cell proliferation and stress responses (Shao et al., 2018). Not surprisingly, perturbations in amino acid metabolism have been identified in many diseases (Abooshahab et al., 2020; Gallagher et al., 2020; Xuan et al., 2020). Similar perturbations are observed in COPD. Plasma or serum amino acid levels are related to energy metabolism and inflammation in COPD, and their disturbances suggest that COPD is a wasting disease with protein degradation (Pouw et al., 1998; Engelen et al., 2007; Ubhi et al., 2012; Ruzsics et al., 2016). Through metabolome analysis of lung tissue, we first discovered the elevated branched chain amino acids (BCAAs) including leucine and isoleucine. BCAAs, as essential amino acids for mammals, are synthesized in bacteria, plants, and fungi but not in animals (Neinast et al., 2019; Bowerman et al., 2020). In lungs of COPD patients, abnormal bacterial diversity has been reported (Bowerman et al., 2020); (Pragman et al., 2012; Ren et al., 2018; Wang et al., 2019), which we speculated might partially interpreted the upregulated BCAAs in lungs. Notably, BCAAs also can be transported into the cells from extracellular medium by amino transporters and then be catabolized in a series of enzymatic reactions (Neinast et al., 2019); (Taylor, 2014). Anyhow, whether the disrupted bacterial diversity, the abnormal expression of amino transporters or key enzymatic associated with BCAAs catabolism contribute to the increased BCAAs needs further exploration.

Moreover, a large proportion of differential metabolites were fatty acyls. Most differential fatty acyls were acylcarnitines; among them, all acylcarnitines associated with l-carnitine were downregulated in the discovery set, and 5 of 6 acylcarnitines were decreased in the replication set. The carnitine shuttle, which refers to l-carnitine, acylcarnitines, CD36 and carnitine acyl transferase Ⅰ/Ⅱ, participates in the translocation of acyl-CoA from the cytoplasm to mitochondria and has an intimate relationship with *β*-oxidation. The imbalance of oxidation/antioxidation contributes to COPD occurrences (Rahman and Adcock, 2006). The decreased acylcarnitines may break the balance of *β*-oxidation, which enhances the development of COPD. As previous studies reported, the progress of the carnitine shuttle was disrupted, and a relatively low level of l-carnitine was identified in the lung, serum and BALF in mice exposed to CS (Agarwal et al., 2014; Conlon et al., 2016). Exogenous carnitine supplementation exhibited antioxidant ability (Vanella et al., 2017). In our study, we validated the abnormality of the carnitine shuttle in the lungs of COPD patients, which might play a role in the development of COPD by being involved in oxidative stress.

COPD is a heterogeneous disease for which few screening biomarkers using omics approaches are available^2^. In our study, we finally screened two overlapping metabolites combining three independent sets as new promising biomarkers. Phytosphingosine alone displayed a strong ability to diagnose COPD with an AUC of 0.8931 (95% CI: 0.8224-0.9639), while l-tryptophan was moderately efficient in discriminating COPD with 0.69 AUC (95% CI: 0.5711-0.8064). The combined diagnostic efficiency of the two metabolites was more optimal (AUC = 0.911, 95% CI: 0.8460-0.9765). This satisfactory diagnostic potential may provide some guidance significance for clinically distinguishing COPD patients from controls in the future. Lung tissue is a direct, effective and accurate approach for evaluating the metabolic profiles of lung of COPD patient. While plasma, a comprehensive reflection of body metabolism, is a convenient, non-invasive but indirect way to explain the metabolic profiles of lung. In our study, phytosphingosine and l-tryptophan were two overlapping differential metabolites in plasma and lung tissue. Since most COPD cases in our study are relatively mild/moderate, we hold that phytosphingosine and l-tryptophan were two indicators for early screening of COPD patients.

Phytosphingosine (PHS) were increased in both plasma and lung tissue. PHS belongs to sphingolipids and exists in plants, yeast, and other mammalian tissues. In yeast and mammalian cells, it can be metabolized to odd-numbered fatty acids and incorporated into glycerophospholipids (Kondo et al., 2014). PHS has been reported to be associated with associated with apoptosis, migration, and inflammation. It enhanced apoptotic cell death in cancer cells through ROS-dependent and -independent AIF release or caspase 8 activation and bax translocation (Park et al., 2003; Park et al., 2005). Takahashi, M., et al. showed that phytosphingosine interacted with CD300b to promote neutrophil recruitment in the way of zymosan-induced, nitric oxide–dependent (Takahashi et al., 2019).Other two studies revealed that PHS and its derivatives ameliorated skin inflammation (Kim et al., 2006; Kim et al., 2014). Furthermore, PHS can activate the endoplasmic reticulum (ER) stress surveillance (ERSU) pathway (Breslow, 2013), which prevented inheritance of stressed ER (Babour et al., 2010; Piña and Niwa, 2015). Reasonably, we speculated PHS may be associated with COPD by affecting apoptosis, inflammation or ER stress. l-tryptophan (L-Trp) is an essential amino acid. Disruptions in l-tryptophan metabolism are reported in several neurological, metabolic, psychiatric, intestinal disorders and cancers, which is also considered as a pharmacological target in clinical practice (Platten et al., 2019; Modoux et al., 2021). The increased L-Trp in lung tissue and decreased L-Trp in plasma have been found in our study and the increased plasma L-Trp is consisting with previous studies (Wendt et al., 2016; Naz et al., 2019). L-Trp can be metabolized by indoleamine 2,3-dioxygenase (IDO), a rate-limiting enzyme in the kynurenine pathway (Cervenka et al., 2017). Interestingly, the reduced sputum IDO activity and expression while the increased serum IDO activity and expression have been reported (Maneechotesuwan et al., 2013; Naz et al., 2019), which may partially explain the divergent change of L-Trp between lung tissue and plasma. Nonetheless, more exploration and underlying mechanisms about the paradoxical changes of L-Trp and IDO in plasma and lung tissue are needed. All in all, PHS and L-Trp are not only the promising metabolite biomarkers to discern COPD from controls but also potential molecular that contributed to the pathogenesis of COPD.

There were several limitations in our study. Firstly, only male patients were enrolled in our study. Women with COPD are less usual than men with COPD but still common (with the prevalence of 8.1% (95% CI [6.8–9.3]) (Fang et al., 2018). The metabolic pattern of lung tissue in women with COPD needs further clarification to provide more awareness of the pathogenesis. Secondly, we did not divide the controls into non-smokers and smokers according to smoking status due to the scarcity of the number of samples. However, we ensured that there was no difference in smoking status between the COPD patients and the controls (Table 1). Further study with a larger of samples should be performed to compare the metabolic profiles of lung tissue in non-smokers, smokers and COPD patients. Finally, considering the instability of untargeted methods, our results also need more studies and targeted metabolomic analysis to further validate.

## Conclusion

In summary, we characterized the metabolic profile of lung tissue from COPD patients. The metabolite profiles of COPD patients were different from those of controls. Additionally, glycerophospholipid metabolism and the valine, leucine and isoleucine biosynthesis pathways were significantly disrupted in COPD patients. Furthermore, we also investigated two metabolites, phytosphingosine and l-tryptophan, as overlapping metabolite biomarkers in three sets. Our results demonstrated that the abnormality of metabolic profiling and the perturbed pathways can help to elucidate the pathological mechanisms underlying COPD, and the screened metabolite biomarkers can effectively discriminate COPD samples from controls. These results may help to establish a foundation for future research attempting to characterize and treat COPD.

## Data Availability

The raw data supporting the conclusion of this article will be made available by the authors, without undue reservation.
