# Supplementary material for: Detection of the Disorders of Glycerophospholipids and Amino Acids Metabolism in Lung Tissue From Male COPD Patients
Source: Front Mol Biosci. 2022 Mar 3;9:839259. doi: 10.3389/fmolb.2022.839259 (PMC8927538; doi:10.3389/fmolb.2022.839259)
Supplement: Supplementary file 1 [file DataSheet1.docx]

**Table S1.** The names of compounds in the Figure S1.

| **names in relative heatmap** | **Compound names** | **Correlation with FVC** | **Correlation with FVC%** | **Correlation with FEV1** | **Correlation with FEV1%** |
| --- | --- | --- | --- | --- | --- |
| **PGs1** | **PG(16:0/20:4)** | **None** | **None** | **None** | **None** |
| **PGs2** | **PG(O-18:0/22:6)** | **None** | **None** | **None** | **None** |
| **PGs3** | **PG(O-18:0/20:5)** | **None** | **None** | **None** | **None** |
| **PGs4** | **PG(O-16:0/22:4)** | **None** | **None** | **None** | **None** |
| **PGs5** | **PG(O-18:0/18:3)** | **None** | **None** | **None** | **None** |
| **PGs6** | **PG(O-16:0/18:2)** | **Negative** | **None** | **None** | **None** |
| **PGs7** | **PG(18:1/0:0)** | **None** | **None** | **None** | **None** |
| **PGs8** | **PG(18:2/0:0)** | **None** | **None** | **None** | **None** |
| **PCs1** | **PC(14:0/20:1)** | **None** | **None** | **None** | **None** |
| **PCs2** | **PC(14:0/20:0)** | **None** | **None** | **None** | **None** |
| **PCs3** | **PC(16:0/20:2)** | **None** | **None** | **None** | **None** |
| **PCs4** | **PC(16:0/18:1)** | **None** | **None** | **None** | **None** |
| **PCs5** | **PC(14:1/22:2)** | **None** | **None** | **Negative** | **None** |
| **PCs6** | **PC(16:0/16:0)** | **None** | **None** | **Negative** | **None** |
| **PCs7** | **PC(14:0/20:2)** | **None** | **None** | **None** | **None** |
| **PCs8** | **PC(14:0/18:1)** | **Negative** | **None** | **None** | **None** |
| **PCs9** | **PC(15:0/18:2)** | **None** | **None** | **None** | **None** |
| **Amino acids1** | **4-Hydroxyproline** | **Negative** | **None** | **None** | **None** |
| **Amino acids2** | **Pyrroline hydroxycarboxylic acid** | **Negative** | **None** | **None** | **None** |
| **Amino acids3** | **L-Glutamic acid** | **Negative** | **None** | **None** | **None** |
| **Amino acids4** | **Homolanthionine** | **Negative** | **None** | **None** | **None** |
| **Amino acids5** | **L-Phenylalanine** | **Negative** | **None** | **None** | **None** |
| **Amino acids6** | **L-Isoleucine** | **Negative** | **None** | **None** | **None** |
| **Amino acids7** | **L-Leucine** | **Negative** | **None** | **None** | **None** |
| **Amino acids8** | **Betaine** | **None** | **None** | **None** | **None** |
| **Amino acids9** | **Proline betaine** | **None** | **Negative** | **None** | **None** |
| **Amino acids10** | **L-Methionine** | **Negative** | **None** | **None** | **None** |
| **Amino acids11** | **Phenylalanylproline** | **Negative** | **None** | **None** | **None** |
| **Amino acids12** | **Creatine** | **Negative** | **None** | **None** | **None** |
| **Amino acids13** | **Pyroglutamine** | **None** | **None** | **None** | **None** |
| **Amino acids14** | **N6-Acetyl-L-lysine** | **Negative** | **None** | **Negative** | **None** |
| **Amino acids15** | **Aminoethoxyacetic acid** | **None** | **None** | **None** | **None** |
| **Amino acids16** | **L-Threonine** | **None** | **None** | **None** | **None** |
| **Amino acids17** | **Pyroglutamic acid** | **None** | **None** | **None** | **None** |
| **Amino acids18** | **N-Acetylserine** | **None** | **None** | **None** | **None** |
| **Amino acids19** | **Pyroglutamylvaline** | **None** | **None** | **None** | **None** |
| **Amino acids20** | **L-Glutamine** | **None** | **None** | **None** | **None** |
| **Amino acids21** | **N-Acryloylglycine** | **None** | **None** | **None** | **None** |
| **Amino acids22** | **L-Asparagine** | **Negative** | **None** | **None** | **None** |
| **Amino acids23** | **Phenylalanyl-Alanine** | **None** | **None** | **None** | **None** |
| **Amino acids24** | **L-Aspartic acid** | **None** | **None** | **None** | **None** |
| **Amino acids25** | **Ne,Ne dimethyllysine** | **Negative** | **None** | **None** | **None** |
| **Amino acids26** | **N6,N6,N6-Trimethyl-L-lysine** | **Negative** | **None** | **None** | **None** |
| **Amino acids27** | **L-Histidine** | **Negative** | **None** | **None** | **None** |
| **Amino acids28** | **L-Lysine** | **Negative** | **None** | **Negative** | **None** |
| **LysoPCs1** | **PE(16:0/P-18:1)** | **None** | **None** | **None** | **None** |
| **LysoPCs2** | **PE(16:0/20:1)** | **None** | **None** | **None** | **None** |
| **Other GPs1** | **PE(14:0/20:1)** | **None** | **None** | **None** | **None** |
| **Other GPs2** | **PI(16:0/22:5)** | **None** | **None** | **None** | **None** |
| **Other GPs3** | **LysoPE(0:0/18:1)** | **None** | **None** | **None** | **None** |
| **Other GPs4** | **LysoPC(18:2/0:0)** | **None** | **None** | **None** | **None** |
| **Other GPs5** | **LysoPC(14:0/0:0)** | **Negative** | **None** | **Negative** | **None** |

**Table S2.** 24 Overlapping differential metabolites between lung tissue and plasma of COPD patients.

| **Metabolites** | **Significance in Discovery set** | **Significance in validation set** | **Significance in Plasma set** |
| --- | --- | --- | --- |
| **L-Tryptophan** | **Yes** | **Yes** | **Yes** |
| **Phytosphingosine** | **Yes** | **Yes** | **Yes** |
| **L-Isoleucine** | **Yes** | **Yes** | **No** |
| **3-hydroxydodecanoyl carnitine** | **Yes** | **Yes** | **No** |
| **L-Carnitine** | **Yes** | **No** | **Yes** |
| **L-Serine** | **Yes** | **No** | **Yes** |
| **Pyruvate** | **Yes** | **No** | **Yes** |
| **L-Histidine** | **Yes** | **No** | **Yes** |
| **C16 Sphinganine** | **Yes** | **No** | **Yes** |
| **SM(d18:1/22:0)** | **Yes** | **No** | **Yes** |
| **3-hexanoyl-NBD Cholesterol** | **Yes** | **No** | **Yes** |
| **C16 Sphingosine** | **Yes** | **No** | **Yes** |
| **Xanthine** | **No** | **Yes** | **Yes** |
| **L-Asparagine** | **No** | **Yes** | **Yes** |
| **L-Methionine** | **No** | **Yes** | **Yes** |
| **Histamine** | **No** | **Yes** | **Yes** |
| **5-Acetylamino-6-formylamino-3-methyluracil** | **No** | **Yes** | **Yes** |
| **Betaine** | **No** | **Yes** | **Yes** |
| **L-Phenylalanine** | **No** | **Yes** | **Yes** |
| **L-Tyrosine** | **No** | **Yes** | **Yes** |
| **Citric acid** | **No** | **Yes** | **Yes** |
| **Pyroglutamic acid** | **No** | **Yes** | **Yes** |
| **Dopamine glucuronide** | **No** | **Yes** | **Yes** |
| **L-Glutamine** | **No** | **Yes** | **Yes** |
